# Making plant science purposeful and relevant to all

**DOI:** 10.1093/jxb/erw201

**Published:** 2016-05-28

**Authors:** Richard Flavell

**Affiliations:** Ceres, Inc., 1535 Rancho Conejo Blvd., Thousand Oaks, CA 91320, USA

**Keywords:** Genome-wide association study (GWAS), GMO, investment, investors, new technologies, roadmap, society.

**Every scientist wants their research to have impact at some level, even if only realized far in the future. But a sense of vision and clear planning are needed today, to inspire everyone from potential investors to schoolchildren contemplating higher education options. Here, the case is made for roadmapping as a critically important way forward for plant science, and a practice that all scientists should take seriously.**

**Figure:**
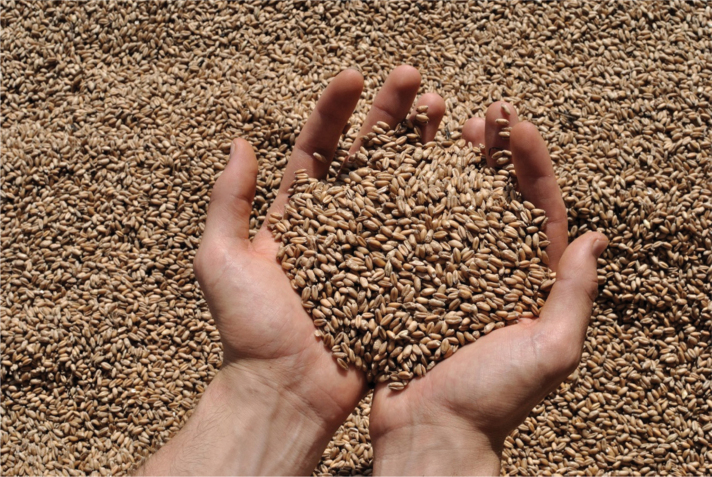
©Fotolia, Sergiogen

The contributions that plants make to the world and societies are well understood in principle. But future contributions require new understandings, investment in research and, in the end, reduction to practice and production. What could these contributions be?

Most of the specific improvements and breakthroughs that might come from research over the next 5, 10, 20 or 50 years and requiring investment are not very visible because they lie embedded in the heads and laboratories of research scientists. Furthermore, scientific progress is unpredictable, diffuse and long-term. Would-be investors in plant science – whether governments, charities, foundations or companies – therefore need to learn from research communities: what are attractive directions and what benefits and breakthroughs can occur in order to consider, justify and prioritize their investments? This is challenging because the output contributions are diverse, and range from new fundamental understanding and concepts to new products and efficiencies in agriculture and environmental management.

## Where should investment be made?

Investors in the public and private sectors want to make beneficial impacts on societies whether through game-changing knowledge, products or services. These options usually come at the end of complex, multidisciplinary research chains. Solving each step in the chain brings great satisfaction and pride to the researchers involved but such steps may have little value to society or investor. Only when the chain has been demonstrated and reduced to practice is real value realized.

Investors are often willing to invest long term towards big achievements – whether in fundamental science or new products, depending on their mission – but rarely in a single next step without understanding its context or place en route to a valuable goal. For example, investors might be willing to invest large sums over a long period aimed at achieving, via a myriad of small consecutive steps, the efficient, cost-effective fixation of gaseous nitrogen in elite cereal varieties, but not in a part of the whole system that has almost no chance of success in being taken forward because it is unconnected. Investors therefore, unlike many scientists, are necessarily strategic in the way they view and prioritize fundamental and applied scientific options.

Investors need to know what is possible, how the goals could be reached, the likelihood of success, approximately how long it might take to realize the goals and the costs involved, however vague they may be. This is what creating roadmaps or long-term ‘business plans’ is all about. They are necessary for investors to make their decisions, but just as importantly they focus the attention of the scientific communities in the public and private sectors to think deeply about what could be possible and how the frontiers of science can be developed to achieve breakthroughs. They are equally applicable to fundamental as well as strategic projects. Such roadmaps do not undermine the Haldane Principle (Report of the Machinery of Government Committee, 1918) that peer review is the best source of judgement on what should be funded and what not.

## Thinking from other fields of research

Defining and prioritizing goals and then creating roadmaps to reach the goals demand brainstorming and creativity beyond current thinking. Some of the most significant outputs from research in plant sciences require breakthroughs in specific technologies, engineering or computing from outside the discipline, and so these have to be recognized, assessed and quantified in making a roadmap. If they are not then some of the most significant achievements of plant science will be missed or not offered in the goals of the roadmap.

Take, for example, the retrospective roadmap for realizing goals in plant breeding by selecting germplasm based on DNA polymorphisms rather than just phenotypes. The early demonstrations of such polymorphisms occurred in the late 1970s when restriction enzymes were first used on plant DNA (Thompson *et al.*, 1983). It was obvious then that a technology and practice like plant breeding that is based on genetic variation would benefit somehow from being able to demonstrate variation in DNA. Some demonstrations of DNA variants in single genes did become useful in breeding in the 1980s, but only in a minor way. Because plant breeding involves creating and surveying variation in detail across all the chromosomes, it was necessary to discover ways of revealing and mapping variation throughout genomes. This was a step in the roadmap that could be envisaged early on from restriction maps of chloroplast DNA, and of other organisms such as yeast and drosophila that came before plant maps. However, the generation and deployment of molecular genetic maps and polymorphisms in plant breeding required the development of high throughput, inexpensive DNA sequencing and chip technologies, as well as extensive computing and algorithm development, from outside plant sciences. Only when such advances were affordable (in the last few years) could genomic selection and genome-wide association study (GWAS) become routinely possible.

Such breakthroughs were the outputs and products from roadmaps and business plans driven towards the markets of human genetics in particular, not plant science. Therefore in creating targets for plant science and roadmaps to define feasible routes forward it is highly desirable to be aware of what technologies are needed. It is also important to know how technologies and breakthroughs in other branches of science, engineering and computing will be the source of breakthroughs in plant science and the delivery of new knowledge, systems and products. Roadmaps should therefore incorporate what can be expected with the technologies of tomorrow, not just the technologies of today.

Similarly, the most exciting goals and roadmaps often require contributions from other institutions many of which may be outside the country or continent. This emphasizes the value of international funding systems to building more progressive roadmaps. Making roadmaps also provokes consideration of what future developments are likely to be acceptable to the public and where technical developments are likely to occur ahead of public support for them. This is an important aspect in plant science given the recent history of GMO foods.

## Real attention to forward planning

There is clearly a need for scientists and investors to understand better the ways in which breakthroughs, not only in plant science but also in other technologies, can and will transform outputs from plant science to benefit science and societies. Part of gaining this understanding can come from deep thinking about and constructing roadmaps. Such processes are part of the discovery process in research and have the added benefit of informing potential investors, thus increasing the chance of gaining substantial and more meaningful investments into science.

Neglecting to foresee important goals and to generate clear plans for how such goals can be reached can only reduce the confidence of investors and society in plant science. Thus they deserve serious attention by the scientific communities and be a routine part of grant writing. Many scientists claim relevance to long-term goals in their grant proposals but in most cases there is little or no credibility in these claims since there is no information given on how the information being proposed would ever be used to achieve the goal. Investors do, of course, recognize the value of training and of supporting employment of scientists and the chances of unforeseen spin offs, but these are often insufficient reasons to invest in a specific piece of research.
